# Soy Isoflavones Ameliorate Metabolic and Immunological Alterations of Ovariectomy in Female Wistar Rats: Antioxidant and Estrogen Sparing Potential

**DOI:** 10.1155/2019/5713606

**Published:** 2019-01-10

**Authors:** Heba M. A. Abdelrazek, Manal M. A. Mahmoud, Hend M. Tag, Sahar M. Greish, Dalia A. Eltamany, Mohammed T. A. Soliman

**Affiliations:** ^1^Department of Physiology, Faculty of Veterinary Medicine, Suez Canal University, Ismailia, Egypt; ^2^Nutrition and Clinical Nutrition Department, Faculty of Veterinary Medicine, Suez Canal University, Ismailia, Egypt; ^3^Department of Biology, Faculty of Sciences and Arts-Khulais, University of Jeddah, PO Box 355, ISIN Code 21-921, Jeddah, Saudi Arabia; ^4^Department of Zoology, Faculty of Sciences, Suez Canal University, PO Box 41522, Egypt; ^5^Department of Physiology, Faculty of Medicine, Suez Canal University, Ismailia, Egypt; ^6^Basic Medical Science Department, Faculty of Dentistry, Badr University in Cairo, Egypt; ^7^Nutrition and Food Science, Home Economic Department, Faculty of Education, Suez Canal University, Ismailia, Egypt; ^8^College of Applied Medical Sciences, Department of Medical Laboratory Sciences, University of Bisha, Saudi Arabia

## Abstract

Hormone replacement therapy (HRT) can alleviate estrogen deficiency symptoms especially during menopause. The present study aimed at investigating the effect of soy isoflavones as HRT on immunological and bone health-related parameters with a special focus on the interactions between immunological status and metabolism. Thirty healthy cyclic female Wistar rats were used in this experiment. Ten females were sham-operated, and 20 females were subjected to ovariectomy. Overiectomized (OVX) female rats were randomly divided into 2 groups: the control group (G1, OVX/casein) was fed a casein-based diet, and the second group (G2, OVX/soy) was fed a high soy isoflavone diet. Both groups were compared to a sham-operated group (G3, sham/casein). Treatments continued for 7 weeks. Feed intake, weight gain, and lymphoid organ relative weights were recorded. Some metabolic, immunological, and bone health-related parameters were measured. Moreover, nitric oxide (NO), malondialdehyde (MDA), and total antioxidant capacity (TAC) were determined. Bone histopathology and immunohistochemistry to estrogen receptor alpha (ER*α*) were done. Feeding soy to OVX females reduced feed intake, weight gain, relative lymphoid organ weight, and T-lymphocytes transformation. Soy isoflavone administration normalized nearly all metabolic and immunological parameters to a level comparable to the sham group via oxidative stress amelioration and bone ER*α* promotion. Soy isoflavones seemed to be good HRT in estrogen deprivation which modulated the appetite, weight gain, lipid profile, proinflammation, and bone turnover.

## 1. Introduction

The estrogen hormone contributes a substantial role in different aspects of body homeostasis and anabolism [1]. It exerts these effects via unexpected regulatory roles on oxidative stress [2], immune function [3], and several metabolic aspects including bone cells as well as adipose tissue [4]. The estrogen hormone gives signals through two main distinguished receptors: estrogen receptor alpha (ER*α*) and estrogen receptor beta (ER*β*) [5]. These receptors are widely spread all over the different body tissues such as reproductive [6], nervous [7], fat [8], liver [9], immune [10], cardiovascular [11], and bone tissues [12].

Estrogen hormone deficiency or depletion has been associated with several metabolic [13] and immunological alterations [14]. These alterations include dyslipidemia, increased appetite, and bone loss [13] that predispose metabolic syndrome along with predisposing autoimmunity and proinflammation [14]. The latter two are characterized by generalized defects in lymphocyte selection and homeostasis along with upregulation in cytokine production [15]. Homeostatic alterations due to estrogen depletion and menopause led researchers to suggest hormone replacement therapy (HRT) to combat their adverse effects.

Several endocrinological, metabolic, and immunological factors as well as oxidative stress were implied in the pathogenesis of these abnormalities along with the interactions with estrogen receptors [16]. Ghrelin is a stomach hormone acting centrally to promote appetite and body weight gain [17]. Moreover, resistin, tumor necrosis factor-alpha (TNF-*α*), and interleukin-6 (IL-6) are adipokine peptides, produced by adipocytes and cytokines that function in metabolic and immunological crosstalk [18]. Both ghrelin and adipokine transcription seemed to be influenced by the estrogen hormone [19]. The calcitonin hormone is beheld as a mediator for estrogen hormone action in bone tissue [20].

Soy isoflavones, a subclass of phytoestrogens, are compounds found in several legumes including soybeans and their products. They include several isoforms divided into four chemical forms: glucoside (genistin, daidzin, and glycitin), aglycone (genistein, daidzein, and glycitein), acetylglucoside (acetylgenistin, acetyldaidzin, and acetylglycitin), and malonylglucoside (malonylgenistin, malonyldaidzin, and malonylglycitin). Isoflavones are considered selective estrogen receptor modulators (SERMs) as they can interact with two estrogen receptor subtypes: ER*α* and ER*β* [21]. They are widely used as dietary supplement in both animals and human diets [9, 22]. Several studies investigated their usage as HRT in case of estrogen depletion [22–26]. Epidemiological data demonstrated the protective effect of isoflavones against age-related chronic diseases [27] and cardiovascular diseases [28] as well as anti-breast cancer effects which were demonstrated by lower incidence in an eastern Asian population where soy predominates diet [29]. Isoflavones also have different biological influences in both animals and humans. These effects include antitumor [30], antimenopausal osteoporosis [31] and antidiabetic, antidyslipidemic [32], and anti-inflammatory effects [33] as well as protective effects against coronary heart diseases [34]. In addition, they exert a myriad of immunological [35, 36], metabolic [37], antioxidant power, and hepatoprotective effects in laboratory animal models [9]. Therefore, the current study aimed at investigating the effects of soy isoflavones as HRT on estrogen deprivation-associated metabolic, immunological, and bone health disturbances in OVX female Wistar rats. The study focused on the possible implication of isoflavones on ghrelin, adipokines, calcitonin, and some immunological parameters in estrogen deprivation conditions.

## 2. Material and Methods

### 2.1. Chemicals

The following chemicals were purchased from Sigma-Aldrich Co., Egypt: Roswell Park Memorial Institute- (RPMI-) 1640 media, trypan blue, fetal calf serum (FCS), phytohaemagglutinin (PHA), tetrazolium dye, and trichloroacetic acid. Ficoll was obtained from Biowest Co., France. Hydrochloric acid, methanol, and acetic acid used in the current study were of HPLC grade and purchased from Fisher Scientific Co., USA. Both genistein and daidzein were obtained from Fujicco Co., Japan, and used as HPLC standards.

### 2.2. Animals, Ovariectomy, and Experimental Procedure

Thirty healthy cyclic female Wistar rats were purchased from a lab animal house at the Faculty of Veterinary Medicine, Suez Canal University. They were 2 months old with a body weight ranging 95-105 g. Rats were kept for 2 weeks for acclimation at natural daylight rhythm and allowed free access to a casein-based diet and water *ad libitum*. All the experimental animals received humane care that accorded with the approved guidelines of the research ethical committee at the Faculty of Veterinary Medicine, Suez Canal University (protocol no. 2018058).

Ten females were sham-operated under 1% thiopental sodium 30 mg/kg intravenous anesthesia. The remaining 20 females were subjected to ovariectomy via midline incision according to the method of Lasota and Danowska-Klonowska [38]. All rats were gavaged with amoxicillin (Amoun Pharmaceutical Co., Egypt) of 10 mg/kg body weight as Ibiamox® in the form of oral suspension for 3 days after surgical intervention [39].

Three weeks after ovariectomy, OVX female rats were randomly divided into 2 groups, 10 rats each. The control group (G1, OVX/casein) was fed a casein-based diet (0% soy that contained 0 *μ*g/g genistein and daidzein as determined by HPLC). The second group (G2, OVX/soy) was fed a high soy isoflavone diet (26.41% soy that contained 1500 *μ*g/g genistein and 800 *μ*g/g daidzein). The sham-operated group was fed a casein-based diet (G3, sham/casein).

All diets were formulated according to NRC [40] to fulfill all the nutritional requirements of adult rats (Table 1) and were given for 7 weeks.

### 2.3. High-Performance Liquid Chromatography (HPLC) Analysis

Dietary isoflavones were subjected to extraction from experimental diets through mixing 1 g of each diet with 20 mL of HCL solution 0.1 mol/L and 80 mL methanol. These ingredients were subjected to sonication for 20 minutes then left for 2 hours at room temperature. The later ingredients were filtered with a filter paper (Clifton, USA). The obtained filtrate was subjected to centrifugation at 10000 rpm for 5 minutes. The supernatant genistein and daidzein were separated and quantified by high-performance liquid chromatography (HPLC) using a reversed-phase column (#50164-U, Sigma-Aldrich Co., Egypt) by using a gradient mobile phase. Solvent A was 0.1% acetic acid, 10% methanol, and 89.9% water; solvent B was 0.1% acetic acid and 99.9% methanol. The solvent B amount was linearly increased from 20% at 0 min to 30% at 2 min to 70% at 30 min. Genistein and daidzein were detected at 260 nm, then they were quantified by comparison with external standards [41].

### 2.4. Feed Intake, Weight Gain, and Lymphoid Organ Weight

Feed intake and body weight gain were recorded/week according to Helmy et al. [39]. Cumulative feed intake and cumulative body weight gain were also calculated. Thymus and spleen were excised from each experimental rat and weighed. The relative thymus and spleen weights were calculated by dividing spleen or thymus weight (g) over body weight (g), then the obtained value was multiplied by 100.

### 2.5. Sampling

At the end of the experimental period, three retro-orbital blood samples were drawn under effect of diethyl ether anesthesia from overnight-fasted rats. The first sample was collected in ethylenediaminetetraacetic acid (EDTA), the second sample in lithium heparin, and the third sample in plain tubes. These samples were used for leukocyte count (total and differential), lymphocytes transformation test (LTT), and serum separation, respectively. Sera were separated from plain tubes, collected, and stored at −80°C. The tibia of each experimental animal was dissected and directly immersed in 10% neutral buffered formalin.

### 2.6. Lipid Profile, Bone Biomarkers, and Ghrelin Level

Serum levels of high-density lipoprotein cholesterol (HDL-C), triglycerides (TG), and total cholesterol (TC) were estimated by the use of enzymatic calorimetric kits (ELITech Diagnostic, France) according to Tietz [42]. Low-density lipoprotein cholesterol (LDL-C) was estimated using an enzymatic calorimetric kit purchased from QAC Co., Spain, according to the manufacturer's protocol. Ionized calcium levels were calculated according to the equation described by Căpriță et al. [43]. *X* = [0.9 + (0.55 × *Y*–0.3 × *Z*)], where *X* is ionized calcium (mg/dL), *Y* is total Ca (mg/dL), and *Z* is albumin (mg/dL).

Both total calcium and total albumin were estimated according to Tietz [42] using enzymatic calorimetric kits (BIOLABO Reagents Co., Maizy, France, and Biodiagnostic, Egypt, respectively). Inorganic phosphorous levels (mg/dL) and alkaline phosphatase activity (ALP) (IU/L) were determined via commercial kits (Biodiagnostic, Egypt, and BIOLABO Reagents Co., Maizy, France, respectively) [42]. Serum ghrelin concentrations were analyzed by the radioimmunoassay method using a standardized rat RIA ghrelin kit (Phoenix Pharmaceuticals Inc., USA). The analytical procedures were done according to the manufacturer's enclosed protocol.

### 2.7. Enzyme-Linked Immunoassay (ELISA), Lipid Peroxidation, and Total Antioxidant Capacity (TAC)

Serum calcitonin and resistin concentrations were determined using commercial rat ELISA kits (Phoenix Pharmaceuticals Inc., USA, and BioVendor Co., Czech, respectively). Serum TNF-*α* (IBL Co., Japan), IL-2, C-reactive protein (CRP) (IBL Co., USA), cyclooxygenase-2 (COX-2) (IBL Co. Japan), and nitric oxide (NO) (Antibodies Online, Germany) levels were assayed using rat ELISA kits. All procedures were done according to the manufacturer's instructions. Malondialdehyde (MDA), a lipid peroxidation biomarker, was calorimetrically assayed using a commercial kit (BioVision, USA) according to Ohkawa et al. [44]. Serum total antioxidant capacity (TAC) was determined via a calorimetric kit (LDN, Germany). All steps were carried out according to the manufacturers' protocol.

### 2.8. Leukocyte Counts and Lymphocytes Transformation Test (LTT)

Blood samples collected in EDTA tubes were subjected to total (TLC) and differential leukocyte (DLC) counts according to Feldman et al. [45]. Freshly obtained lithium heparinized blood samples were immediately transferred to the laboratory in ice bags. Lymphocytes were separated using Ficoll at 2400 rpm for 40 minutes in a cooling centrifuge. Separated lymphocytes were washed and suspended in RPMI-1640 medium. The viable lymphocyte cell count was adjusted 2 × 10^6^/mL using trypan blue and a hemocytometer slide [46]. The viable lymphocytes were suspended in RPMI-1640 medium supplemented with 10% FCS. Lymphocytes were assayed for their transformation ability against PHA mitogen (15 *μ*g/mL) using methyl thiazolyl tetrazolium (MTT) staining procedures [47].

### 2.9. Histopathology and Immunohistochemistry (IHC)

Formalin-fixed tibia were subjected to decalcification in 5% trichloroacetic acid for 20 days then dehydrated and stained with H&E. All histopathological procedures were performed according to Bancroft and Gamble [48]. Paraffin-embedded tibia were subjected to immunohistochemistry (IHC) using a primary antibody for ER*α* (Thermo Scientific Co., UK) according to the methodology of Helmy et al. [39]. The percentages of the IHC-stained area (IHC area %) were obtained using ImageJ software according to Elgawish et al. [49].

### 2.10. Statistical Analysis

The obtained data were expressed as means ± SEM and subjected to analysis by one-way ANOVA using SPSS (IBM SPSS Statistics, version 22, USA). Differences among means were tested at the 5% probability level using Duncan's multiple range test.

## 3. Results

### 3.1. Feed Intake, Weight Gain, and Lymphoid Organ Weight

Cumulative feed intake significantly (*p* < 0.05) declined in G2 (OVX/soy) compared to G1 (OVX/casein). The sham-operated group (G3) showed a significant reduction in cumulative feed intake than did both ovarictomized groups (G1 and G2). Cumulative weight gains significantly (*p* < 0.05) reduced in G2 (OVX/soy) compared with those in G1 (OVX/casein). However, there was no difference (*p* > 0.05) observed between the OVX/soy group and the sham-operated one (Table 2). The relative weights of thymus reduced (*p* < 0.05) in soy-fed OVX females than in casein-fed OVX females and sham ones. Spleen weights showed nonsignificant alterations among the tested groups (Table 2).

### 3.2. Lipid Profile, Bone Biomarkers, and Ghrelin Level

Table 3 shows improvement in lipid profile in OVX females fed a soy diet. HDL-C significantly (*p* < 0.05) improved in the soy OVX group than in the casein OVX group. There was no significant difference in HDL-C between G2 and the sham-operated group. Serum levels of LDL-C, TG, and TC declined significantly (*p* < 0.05) in G2 than in G1. However, nonsignificant changes were observed between G2 and G3. Serum-ionized Ca and phosphorus levels were reduced (*p* < 0.05), while ALP activity was elevated (*p* < 0.05) in the soy OVX group than in the casein OVX group (Table 3). Ghrelin hormone level significantly (*p* < 0.05) reduced in G2 when compared to G1. No significant difference was observed between G2 and G3 (Table 3).

### 3.3. Enzyme-Linked Immunoassay (ELISA), Lipid Peroxidation, and Total Antioxidant Capacity (TAC)

Soy feeding to OVX female rats exhibited an elevated (*p* < 0.05) calcitonin level compared to casein-fed ones nearly equal to the sham group level. Resistin level revealed a significant (*p* < 0.05) reduction in G2 than in G1; however, there was no significant difference observed between G2 and G3 (Table 3). Serum TNF-*α*, IL-2, CRP, COX-2, and NO significantly (*p* < 0.05) reduced in G2 than in G1 while their values nonsignificantly differed with G3 (Table 4). The level of MDA revealed a highly significant (*p* < 0.01) elevation in G1 compared to G2 and G3. The level of TAC revealed a significant (*p* < 0.01) promotion in G2 than in G1. Both MDA and TAC showed a nonsignificant variation between G2 and G3 (Table 4).

### 3.4. Leukocyte Counts and Lymphocytes Transformation Test (LTT)

**Figure 1:**
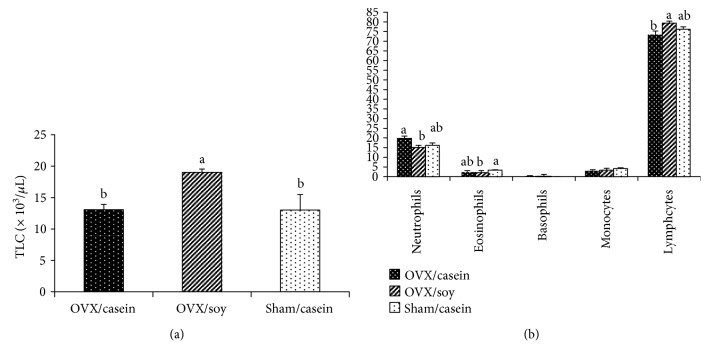
Effect of soy isoflavones on total (a) and differential leukocyte count (b) among experimental groups. Bars with different superscripts are significantly different at *p* < 0.05; values are presented as means ± SEM.

Blood TLC was significantly (*p* < 0.05) elevated in the soy OVX group than in the casein OVX group and sham-operated ones (Figure 1(a)). Neutrophils % showed a significant (*p* < 0.05) reduction in G2 than in G1; however, sham-operated rats in G3 did not show any significant variation when compared with those of G1 and G2. Eosinophils % demonstrated a significant (*p* < 0.05) decline in G2 than in G3. Casein-fed OVX rats showed nonsignificant changes when compared with soy OVX and sham-operated ones. Basophils % and monocytes % revealed nonsignificant changes among tested groups. Lymphocytes % showed a significant (*p* < 0.05) increment in G2 than in G1 rats. Sham-operated rats in G3 did not reveal any significant change in lymphocytes % as compared with those of G1 and G2 (Figure 1(b)). Lymphocytes transformation exhibited significant (*p* < 0.05) suppression in G2 than in both G1 and G3. A nonsignificant difference was observed between G1 and G3 (Table 4).

### 3.5. Histopathology and Immunohistochemistry

**Figure 2:**
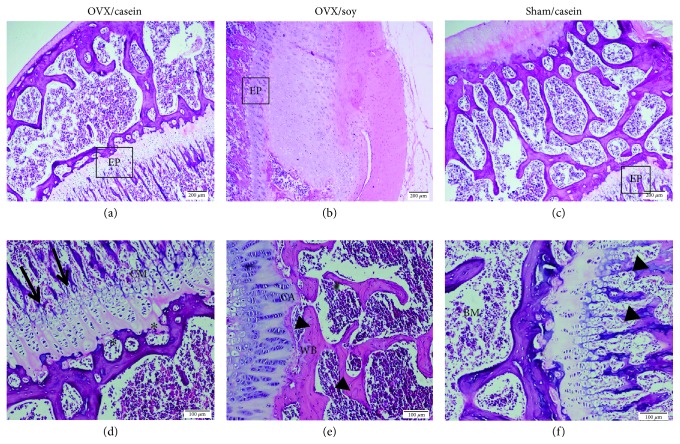
Photomicrographs of epiphyseal bone and epiphyseal plate of rat tibia stained with H&E. (a, b, c) Scale bar: 200 *μ*m. (d, e, f) Scale bar: 100 *μ*m. (a, d) OVX/casein group, (b, e) OVX/soy group, and (c, f) sham/casein group. BM: bone marrow; WB: woven bone; CM: zone of cartilage maturation and hypertrophy; CA: zone of cartilage ossification between cartilage and the zone of bone deposition. Arrows represent resorbed bone trabeculae. Head arrows represent well-developed bone trabeculae. Asterisks represent resorbed bone trabeculae.

**Figure 3:**
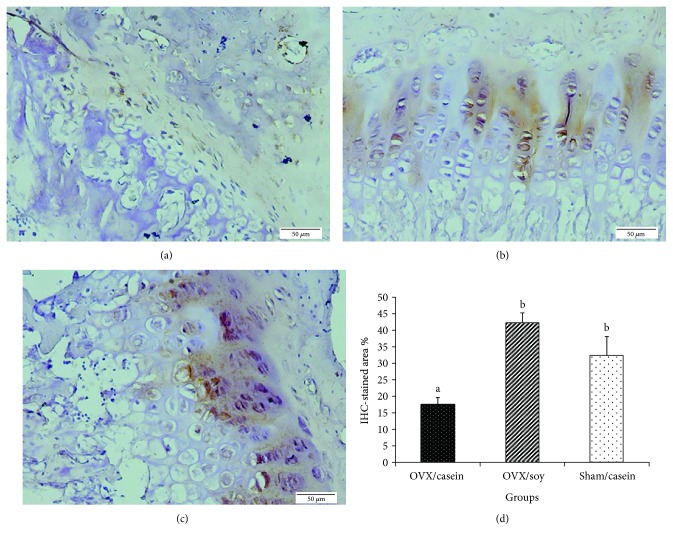
A photomicrograph of a section from tibia bone showing estrogen receptor–*α* expression. (a) OVX/casein group, (b) OVX/soy group, (c) sham/casein group, and (d) a bar chart showing a comparison of the three groups in the estrogen receptor-*α* IHC-stained area % (IHC stain, scale bar: 50 *μ*m).

A histological examination of tibia from the treated and control groups are shown in Figure 2. After 7 weeks of ovariectomy, females fed a casein-based diet revealed changes in the growth of epiphyseal plate structure. The architecture of the growth plate showed fewer proliferative chondroblastic cells with few and thinner trabeculae as compared with sham and ovarictomized females fed a high soy isoflavone diet (Figures 2(a) and 2(d)). Also, the zone of cartilage ossification appeared to be resorbed. After 49 days of treatment with soy (Figures 2(b) and 3(e)), the tibia morphology was almost identical to that of the intact sham-operated group (Figures 2(c) and 2(f)). The epiphyseal plate was well developed and contained a typical arrangement of a proliferative, chondroblastic pattern. Besides, newly formed woven bones were observed which are a microscopic evidence of new bone formation, as well as well-developed bone trabeculae and ossification between cartilage and the zone of bone deposition. Soy isoflavones significantly (*p* < 0.001) increased the IHC-stained area % than the casein-fed OVX group, while there was no significant difference observed between G2 and G3 (Figure 3).

## 4. Discussion

Metabolic and immunological disorders are common fate to estrogen deficiency or deprivation. In this case, the research for hormone replacement supplement is mandatory [25]. The usage of soy isoflavones as HRT to alleviate adverse effects of estrogen deficiency was tested in the present study. The level of soy isoflavones that was ingested by OVX females at the present study falls within the same range of soy ingested by the Asian population. Those people consumed 20 to 50 g of soy daily. Dietary isoflavone analysis by HPLC in the current study revealed 800 *μ*g/g daidzein and 1500 *μ*g/g genistein that were together equal to the Asian people intake which was estimated to be about 20 to 80 mg of phytoestrogens/day [50]. Moreover, dietary murine genistein equivalent to 1000 or 1500 *μ*g/g was reported to produce serum genistein concentrations that matched the physiological range of humans under dietary phytoestrogens regimes [51]. Dietary genistein at 1500 *μ*g/g was also reported by some publications to have immunological [52] and antilipogenic effects [53].

Cumulative feed intake and weight gains significantly declined in OVX females fed soy than casein-fed OVX ones to a level comparable to the sham-operated group. On the parallel side, ghrelin hormone followed the same trend of decrement. The possible explanation is that the absence of ovarian estrogen in OVX females led to a downregulation of hypothalamic estrogen receptors (ERs) restricting feed intake and modulating energy expenditure [54]. Hence, feed intake and body weight gain increased. Feeding soy isoflavones, as SERM, could result in overregulation of hypothalamic ERs that restricted feed intake and subsequent body weight gains to a level comparable to sham-operated females with intact ovaries. Moreover, soy isoflavones dramatically reduced the orexigenic ghrelin hormone level; therefore, it controlled the appetite-inducing action of ovariectomy [16] in this group. Our results were in partial agreement with Cederroth et al. [37] who demonstrated reduced feed intake and body weight gain without change in ghrelin hormone level in soy-fed mice.

Dietary soy had direct influences on lipid metabolism as it diminished TG, TC, and LDL-C and promoted HDL-C. These results were consistent with previous records of Tolba [55] and Yousefinejad et al. [56]. The observed hypolipidemic effect in G2 may be ascribed to the reduction in ghrelin hormone level which is considered a potent growth hormone secretagogue [57]. Ghrelin can promote white adipose tissue lipogenesis through a hypothalamic-mediated mechanism [58]. Thus, its reduction led to the observed hypolipidemia. Furthermore, soy isoflavones have the ability to decrease intestinal cholesterol absorption via increase in bile acid excretion [59]. Also, the capacity of soy isoflavones to decrease the lipid profile is related to AMPK activation which enhances fatty acid oxidation in liver and adipocytes [37].

Thymus relative weights significantly reduced in the soy OVX group than in OVX/casein and sham ones. However, splenic relative weights showed nonsignificant alterations. Our results coincided with those of Kakehashi et al. [60], Nishide et al. [61], and Ebaid et al. [36]. It is not surprising to find a similarity between dietary isoflavone exposure and estrogen's hormone action in mediating thymic atrophy [62] as well as suppression of LTT [63]. Isoflavones can overregulate and bind ERs especially ER*α* that exerts a potential restricting role to T-lymphocytes proliferation [64]. Soy genistein can also inhibit protein tyrosine kinases that subsequently suppress several white blood cells signaling cascades especially IL-2. These signaling cascades are involved in thymocytes and T-lymphocytes differentiation as well as their proliferation [65].

The dietary soy isoflavones significantly increased TLC with lymphocytosis at the expense of neutrophils than casein-fed rats. Current results were parallel to records of Jenkins et al. [66] and Cheng et al. [67]. Soy isoflavones mimicked estrogen in this group, therefore causing downregulation of adhesion molecules and chemokines that altered leukocytes recruitment and chemotaxis and thus exerting their anti-inflammatory action [68]. The elevation of lymphocytes percent on expense of neutrophils augmented the compensative effect of soy isoflavones to oxidative stress induced by gonadal removal. The relation between neutrophils and lymphocytes was used as indicator for inflammation [69], oxidative stress [70], and cortisol production [71]. The eosinopenia that happened in the soy-treated group was suggestive for the antiallergic effect of isoflavones. Administration of dietary soy isoflavones seemed to resemble estrogen action that could regulate eosinophils recruitment and cause their degranulation [72].

Ovariectomy accelerated oxidative stress that is demonstrated by increased NO, as an oxidative stress biomarker, and lipid peroxidation (MDA) with reduction in TAC that was normalized to the sham group level in the soy group. Our results were similar to those of Wang and Wu [73], Tang et al. [74], and Onuegbu et al. [75]. Oxidative stress is a casual factor for several metabolic and immunological disorders [76]. Lipid profile abnormalities observed in G1 could be the principle cause for lipid peroxidation and generation of excess reactive oxygen species (ROS) [9]. Soy isoflavone phenolic rings can act directly via free radical scavenging or indirectly via modulation of the pro- and antioxidant intracellular enzyme expression [77]. Furthermore, soy isoflavones can reduce inducible NO synthase enzyme and hence affect all physiological pathways that NO is involved in [78]. One of these pathways is leukocyte chemotactic response [79] which is manifested here by increased TLC in soy-treated rats.

Moreover, the function of NO as an intracellular messenger in chemokine signaling pathways [80] decreased and was manifested by decreasing levels of TNF-*α* and IL-2 in the soy OVX group to a level comparable to sham ones. These decrements were in accordance with results of Shalaby and Elgawish [81], Azadbakht et al. [26], and Gaffer et al. [35]. The scavenging effect of soy isoflavones to ROS which was demonstrated by restoration of TAC as well as reduction in MDA could entangle ROS-mediated NF-*κ*B/TNF-*α* signaling activation [82]. In addition, soy isoflavones could promote ER expression that has a reciprocal antagonism NF-*κ*B activity [83]. The latter promotes TNF-*α* production [82]. Moreover, ER promotion by soy isoflavones [65] could inhibit protein tyrosine kinase and topoisomerase II [63, 84]. These two enzymes are essential for IL-2 production. These results could briefly explain the decrease in LTT values where IL-2 plays a substantial role in T-cell proliferation in an autocrine manner [65, 85].

Our study demonstrated a significant reduction in serum resistin level in the soy-fed OVX group to a level around to that of sham-operated rats. Current results were harmonized with those of Chen et al. [86] and Zhang et al. [87]. Resistin is produced from adipose tissue and induces an inflammatory activation, with the production of TNF-*α* by macrophages through the NF-*κ*B pathway [88]. These findings augmented the idea that resistin level is regulated and correlated to the level of TNF-*α* [89] as well as its pro-inflammatory potential [90]. This decrease in resistin levels could be implied for the effect of soy isoflavones on peroxisome proliferator-activated receptors (PPARs) in endothelial and mononuclear cells [91]. PPAR is a major factor involved in de novo fatty acid synthesis, adipocyte differentiation, and lipid accumulation [92]. Furthermore, PPARs cause resistin repression through direct binding to the resistin promoter [93]. The normalization of ovariectomy-induced resistin elevation by soy feeding was augmented with the reduction of body weight gain in such group. This was suggestive for the restoration of low abdominal fat mass, normal glucose metabolism, and insulin sensitivity that were impaired after ovariectomy (data not shown). The resistin hormone had a positive correlation with CRP level that is considered an inflammatory biomarker [94]; thus, CRP was significantly reduced in the soy OVX group than in the casein-fed OVX one. Decreased body weight gain was also attributed to CRP reduction due to a strong correlation between these two parameters [95]. The antioxidant potential of soy isoflavone polyphenolic ring had a negative influence on serum CRP as antioxidants negatively influence CRP production [96]. Furthermore, it is logic to find the downregulation of CRP level together with reduced TNF-*α* and IL-2 where CRP is produced by hepatic cells in response to such cytokines [97]. The decrement in CRP after soy isoflavone treatment was in harmony with results of Fanti et al. [98] and Jin et al. [99].

COX-2 is a critical proinflammatory enzyme that converts arachidonic acid to prostaglandins that have been implicated in pain and inflammation [100]. The reduction in COX-2 level in G2 could explain the anti-inflammatory effect of soy isoflavones in OVX rats. These results were generally consistent with those reported by Hooshmand et al. [101], Valles et al. [102], and Khan et al. [103]. Isoflavone modulation to estrogen receptors, as SERM, is involved in regulation of COX-2 production and its bioactivity [104]. Isoflavones especially genistein had a repressing action on NF-*κ*B that in turn represses COX-2 genesis [101, 105]. Moreover, the antioxidant power of soy isoflavones has an inhibitory effect on the activation of protein kinase C [106] and activator protein-1 [107] that play a role in COX-2 promoter activity [100].

Ovariectomy hastens bone turnover, manifested by increased levels of ionized Ca^2+^ and phosphorous while decreased ALP activity denoted osteoblast activity. It also reduced chondroblastic cell proliferation with fewer and thinner trabeculae as well as resorption in the zone of cartilage ossification. These changes were also accompanied with reduced calcitonin hormone level that is known to be decreased in gonadal hormone deficiency [108]. The reduction in bone mass was attributed to depletion of ovarian estrogen and its bone receptors as observed in IHC. Soy feeding to OVX females promoted an IHC-stained area % of ER*α*, which decreased osteoclast activity while promoting osteoblast activity [109] through upregulation of calcitonin hormone level [20]. Therefore, they decreased serum-ionized Ca^2+^ and phosphorous levels favoring their deposition in the bone matrix as well as elevated ALP activity [110] to a level near the sham group. Elevated ALP activity may indicate active bone formation, as it is a byproduct of osteoblast activity [111]. In addition, the reduction in ghrelin level in G2 could be related to the decreased bone density as it directly influenced osteoblast function or indirectly via the growth hormone insulin-like growth factor axis [112]. Our results concerning bone restoration, calcitonin, ionized Ca^2+^, phosphorous, and ALP activity were in agreement with Zhong and Yamaguchi [113], Lee et al. [114], Wafay et al. [115], Nurrochmad et al. [116], and Hassan et al. [117]. Both TNF-*α* and IL-2 are present in the bone microenvironment, and their levels are indicative for bone health. Elevation of such cytokines led to progression of bone turnover and resorption [118]. The secretion of such cytokines was inhibited through the estrogenic influence of soy isoflavones. Their estrogenic action is attributed to reduction in bone turnover and increased osteoblastic activity [119] of OVX females in order to restore estrogen depletion. The estrogenic effect of soy isoflavones seemed to be overlapped with its ROS scavenging effect at the bone level. The antioxidant power of isoflavone polyphenols was able to scavenge excessive nitric oxide and MDA as well as promotion of TAC. These effects neutralized the ovariectomy of ROS that incriminated in the pathogenesis of bone loss excessive activity of osteoclasts and bone mineralization [120]. Therefore, a histopathological picture of the Soy/OVX group showed ossification improvements by presenting a well-developed epiphyseal plate that contained proliferative chondroblasts, besides the presence of newly formed woven bone and well-developed bone trabeculae.

## 5. Conclusion

Ovariectomy as a model for estrogen depletion resulted in a myriad of metabolic alterations and bone turnover that were promoted by excessive ROS production. Feeding soy isoflavones improved the lipid profile and subsequently the antioxidant reserve that exerted an anti-inflammatory effect and improved bone mineralization via the calcitonin hormone. Moreover, soy feeding restored ER deficiency that is implicated in appetite promotion, proinflammation, and bone loss, thus overcoming these deleterious effects.

## Figures and Tables

**Table 1:** Diet composition.

Ingredients	Control (%)	High isoflavones (%)
Yellow corn	40.59	35.04
Corn gluten	15.00	—
Soybean^∗^	—	26.41
Casein	5.00	5. 00
Sucrose	22.43	22.32
Starch	7.63	4.16
Cellulose	1.30	—
Corn oil	5.00	—
Soybean oil	—	5.00
Methionine	0.30	0.43
Lysine	0.26	—
Tryptophan	0.39	—
Ground limestone	0.58	0.70
Dicalcium phosphate	1.09	0.57
Common salt	0.13	0.13
Premix^∗∗^	0.30	0.30
Total	100.00	100.00
*Calculated values*		
CP %^∗∗∗^	17.11	17.11
ME (kcal/kg)	3708.26	3703.05
C/P ratio	216.70	216.50
Ca	0.50	0.50
P	0.30	0.30
*Dietary HPLC analysis data*		
Genistein (*μ*g/g)	0	1500
Daidzein	0	800

^∗^Soybean was autoclaved at 110°C for 30 minutes according to Westfall and Hauge [121] to inactivate the trypsin inhibitor, tannins, saponins, phytate, protease inhibitors, lectins, and goitrogens. ^∗∗^Each 3 kg contains the following vitamins and minerals: vit. A 12 mIU, vit. D_3_ 2 mIU, vit. E 1000 mg, vit. k_3_ 1000 mg, vit. B_1_ 1000 mg, vit. B_2_ 5000 mg, vit. B_6_ 1500 mg, vit. B_12_ 10 mg, biotin 50 mg, pantothenic acid 10000 mg, nicotinic acid 30000 mg, folic acid 1000 mg, manganese 60000 mg, zinc 50000 mg, iron 30000 mg, copper 4000 mg, iodine 300 mg, selenium 100 mg, cobalt 100 mg, and carrier (CaCO_3_) to 3 kg (Golden premix- Selim Pharm Elasher, Egypt.). ^∗∗∗^Analyzed according to Helrick [122].

**Table 2:** Effect of soy isoflavones on feed intake (g/day/female), cumulative feed intake (g/female), weight gain (g/week), cumulative weight gain (g), and relative thymus and spleen weight (g%) among experimental groups.

Parameters		G1 (OVX/casein)	G2 (OVX/soy)	G3 (sham/casein)
Feed intake (g/day/female)	1st week	19.34 ± 1.11^a^	17.20 ± 1.17^a^	12.57 ± 0.68^b^
	2nd week	18.75 ± 1.20^a^	12.92 ± 0.86^b^	11.65 ± 1.15^b^
	3rd week	17.40 ± 0.67^a^	14.63 ± 0.56^ab^	12.87 ± 1.5 1^b^
	4th week	16.42 ± 0.84^a^	16.22 ± 1.03^a^	11.77 ± 1.33^b^
	5th week	21.93 ± 2.77^a^	18.51 ± 0.80^ab^	13.21 ± 1.39^b^
	6th week	21.97 ± 3.44^a^	18.37 ± 0.95^ab^	13.80 ± 1.42^b^
	7th week	21.33 ± 1.36^a^	16.68 ± 3.14^a^	9.75 ± 0.57^b^
Cumulative feed intake (g/female)		956.22 ± 6.15^a^	801.47 ± 5.41^b^	599.48 ± 11.55^c^

Weight gain (g/week)	2nd week	19.70 ± 1.08^a^	11.73 ± 1.91^b^	9.47 ± 1.28^b^
	3rd week	10.45 ± 1.58^a^	10.65 ± 0.88^a^	8.90 ± 1.10^a^
	4th week	12.19 ± 1.11^a^	8.02 ± 0.80^b^	5.37 ± 0.98^b^
	5th week	7.08 ± 1.12^a^	6.73 ± 0.63^a^	5.73 ± 0.37^a^
	6th week	6.32 ± 0.81^a^	3.62 ± 0.40^b^	2.97 ± 0.49^b^
	7th week	5.33 ± 0.96^a^	1.05 ± 0.52^b^	1.07 ± 0.47^b^
Cumulative weight gain (g)		44.12 ± 5.13^a^	31.60 ± 5.01^b^	23.45 ± 2.39^b^

Relative weight (g%)	Thymus	0.35 ± 0.04^a^	0.16 ± 0.02^b^	0.32 ± 0.05^a^
	Spleen	0.58 ± 0.04^a^	0.50 ± 0.09^a^	0.45 ± 0.06^a^

^a-c^Means in the same row with different superscripts are significantly different (*p* < 0.05); values are presented as means ± SEM.

**Table 3:** Effect of soy isoflavones on serum lipid profile, ghrelin, calcitonin, ionized calcium, inorganic phosphorus, and alkaline phosphatase (ALP) among experimental groups.

Parameters	G1 (OVX/casein)	G2 (OVX/soy)	G3 (sham/soy)
HDL-C (mg/dL)	10.19 ± 0.41^a^	12.58 ± 0.44^b^	14.01 ± 0.20^b^
LDL-C (mg/dL)	65.72 ± 1.96^a^	55.08 ± 0.94^b^	51.31 ± 4.72^b^
TG (mg/dL)	109.80 ± 4.85^a^	83.65 ± 8.49^b^	83.15 ± 2.21^b^
TC (mg/dL)	66.85 ± 2.41^a^	58.32 ± 1.27^b^	55.89 ± 2.87^b^
Ghrelin (pg/mL)	545.80 ± 3.49^a^	370.80 ± 6.55^b^	369.50 ± 13.87^b^
Calcitonin (pg/mL)	172.46 ± 5.98^b^	249.90 ± 4.19^a^	252.83 ± 2.50^a^
Resistin (ng/mL)	0.87 ± 0.05^a^	0.59 ± 0.04^b^	0.61 ± 0.04^b^
Ionized Ca^+2^ (mg/dL)	6.77 ± 0.35^a^	5.77 ± 0.19^b^	5.19 ± 0.30^b^
Phosphorus (mg/dL)	6.04 ± 0.12^a^	5.10 ± 0.33^b^	4.98 ± 0.37^b^
ALP (IU/L)	126.00 ± 5.12^b^	190.80 ± 12.88^a^	179.3 ± 3.57^a^

^a-b^Means in the same row with different superscripts are significantly different (*p* < 0.05); values are presented as means ± SEM.

**Table 4:** Effect of soy isoflavones on lymphocyte transformation test (LTT), nitric oxide (NO), total antioxidant capacity (TAC), malondialdehyde (MDA), and inflammatory mediators among experimental groups.

Parameters	G1 (OVX/casein)	G2 (OVX/soy)	G3 (sham/soy)
LTT	0.65 ± 0.06^a^	0.27 ± 0.04^b^	0.51 ± 0.06^a^
NO (*μ*M/L)	29.41 ± 0.85^a^	21.29 ± 0.91^b^	21.84 ± 0.66^b^
TAC (mM/L)	0.34 ± 0.12^a^	0.82 ± 0.08^b^	0.72 ± 0.04^b^
MDA (nM/L)	1.97 ± 0.13^a^	1.61 ± 0.07^b^	1.53 ± 0.09^b^
TNF-alpha (pg/mL)	8.99 ± 0.30^a^	5.73 ± 0.56^b^	5.25 ± 0.46^b^
IL-2 (pg/mL)	5.74 ± 0.54^a^	3.54 ± 0.27^b^	3.39 ± 0.39^b^
COX-2 (ng/L)	9.75 ± 0.42^a^	6.70 ± 0.59^b^	7.50 ± 0.44^b^
CRP (mg/L)	1.56 ± 0.03^a^	1.09 ± 0.07^b^	1.04 ± 0.08^b^

^a-b^Means in the same row with different superscripts are significantly different (*p* ≤ 0.05); values are presented as means ± SEM.

## Data Availability

No data were used to support this study.

## Supplementary Materials

Supplementary Materials Supplementary material contains a graphical abstract that describes the experimental design and results with implications of these results.
