# Supplementary figures and images for: Soy Isoflavones Ameliorate Metabolic and Immunological Alterations of Ovariectomy in Female Wistar Rats: Antioxidant and Estrogen Sparing Potential
Source: Oxid Med Cell Longev. 2019 Jan 10;2019:5713606. doi: 10.1155/2019/5713606 (PMC6348823; doi:10.1155/2019/5713606)

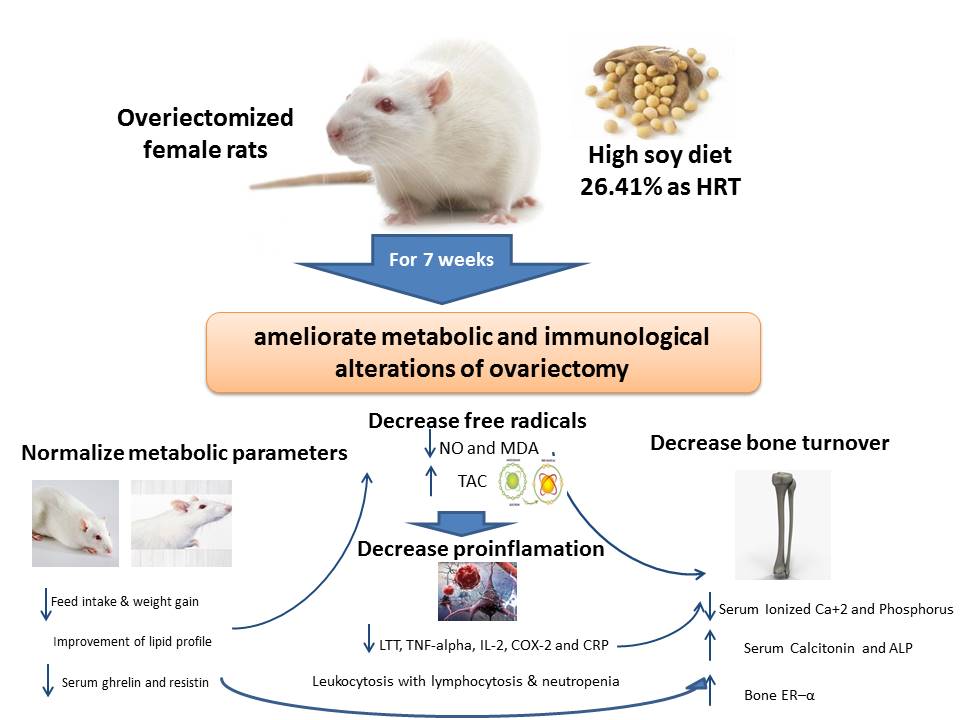

Supplement: Supplementary Materials — Supplementary material contains a graphical abstract that describes the experimental design and results with implications of these results. [file 5713606.f1.jpg]
